# Single-Particle Photothermal Circular Dichroism and Photothermal Magnetic Circular Dichroism Microscopy

**DOI:** 10.1021/acs.nanolett.4c00448

**Published:** 2024-04-05

**Authors:** Subhasis Adhikari, Maria V. Efremova, Patrick Spaeth, Bert Koopmans, Reinoud Lavrijsen, Michel Orrit

**Affiliations:** † Huygens-Kamerlingh Onnes Laboratory, Leiden University, 2300 RA Leiden, The Netherlands; ‡ Department of Applied Physics and Science Education, Eindhoven University of Technology, P.O. Box 513, 5600 MB Eindhoven, The Netherlands; § Department of Sustainable Energy Materials, AMOLF; Science Park 104, 1098 XG Amsterdam, The Netherlands

**Keywords:** Chirality, chiral plasmonics, magnetism, metal nanoparticles, magnetic nanoparticles, magneto-optical Kerr effect, nanotechnology, magnetization switching

## Abstract

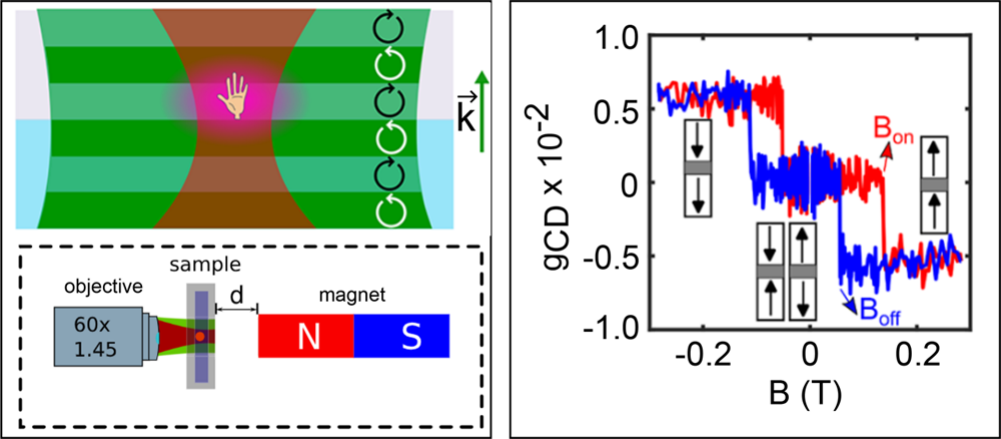

Recent advances in single-particle photothermal circular dichroism (PT CD) and photothermal magnetic circular dichroism (PT MCD) microscopy have shown strong promise for diverse applications in chirality and magnetism. Photothermal circular dichroism microscopy measures direct differential absorption of left- and right-circularly polarized light by a chiral nanoobject and thus can measure a pure circular dichroism signal, which is free from the contribution of circular birefringence and linear dichroism. Photothermal magnetic circular dichroism, which is based on the polar magneto-optical Kerr effect, can probe the magnetic properties of a single nanoparticle (of sizes down to 20 nm) optically. Single-particle measurements enable studies of the spatiotemporal heterogeneity of magnetism at the nanoscale. Both PT CD and PT MCD have already found applications in chiral plasmonics and magnetic nanomaterials. Most importantly, the advent of these microscopic techniques opens possibilities for many novel applications in biology and nanomaterial science.

Most biomolecules in our body are chiral, and their biomolecular interactions depend on their handedness. A molecule is chiral when it cannot be superimposed onto its mirror image. The molecule and its mirror image are called stereoisomers or enantiomers. They have the same chemical formula, but their (bio)chemical functions depend on their handedness. It is important for the pharmaceutical industry to distinguish enantiomers because one enantiomer of a drug can be beneficial in curing a disease, while the other enantiomer can be harmful. In a standard way, the drug industry uses circular dichroism (CD) spectroscopy for enantioselective detection of chiral molecules. CD is defined as the differential absorption of left- and right-circularly polarized light by a chiral object. CD spectra are specific to a certain conformation of a biomolecule, and the sign of CD depends on the handedness of an enantiomer. CD spectroscopy is thus a powerful method for obtaining information about the conformation of a protein molecule. However, commercial CD spectrometers can measure only ensembles of molecules and have a poor spatial resolution. In the past decade, there has been a strong growth in the development of single-particle CD spectroscopy.^1^ Circular dichroism of a single chiral nanoparticle can be detected by measuring a difference in extinction,^2^ scattering,^3^ or absorption^4,5^ between left- and right-circularly polarized light. However, extinction and scattering entail both the imaginary and real parts of the dielectric function, whereas CD is related to only the imaginary part of the dielectric function. Therefore, care needs to be taken to distinguish pure CD from CB (circular birefringence, also called optical rotatory dispersion, ORD) of a chiral object.^6^ A true CD can be obtained by the direct measurement of the differential absorption of a chiral object. Photothermal circular dichroism (PT CD) microscopy based on photothermal (PT) microscopy is able to measure the direct differential absorption of left- and right-circularly polarized light. The PT CD concept was initially demonstrated as thermal lens CD spectroscopy of ensembles of diffusing molecules by Kitamori’s group.^7^ Later, a theoretical work was reported by Kong et al.^8^ Recently, some of us^4,9^ have demonstrated the detection of single-particle PT CD signals with a high sensitivity, down to a g_CD_-factor of a few 10^–4^.

Optical imaging of magnetization is an important tool for understanding the magnetic properties of nanomaterials and their applications to biological systems. Magneto-optical phenomena such as the Faraday effect and Kerr effect are well-known light-matter interactions with magnetic materials. Recent advances in the ultrafast manipulation of magnetization with light can be applied to data storage and spintronics. Magnetic nanoparticles have shown great potential for applications in biology, because of their biocompatibility. For example, magnetic hyperthermia using magnetic nanoparticles can be used for cancer treatment. Currently, most existing methods either lack spatial resolution or demand complicated and expensive experimental designs. For example, superconducting quantum interference devices (SQUIDs), magneto-optical Kerr microscopes, or conventional magnetic circular dichroism (MCD) microscopes require ensembles of particles for sensitive magnetic measurements. However, the magnetic properties of nanoparticles depend on their size, shape, and orientation. Single-particle X-ray magnetic CD (XMCD)^10^ of iron, cobalt, and nickel nanoparticles has shown heterogeneous magnetic properties of individual nanoparticles depending on their physical properties. XMCD requires expensive X-ray synchrotron sources in dedicated facilities, which therefore limits throughput and applicability. Advanced scanning SQUID devices reach spatial resolution and sensitivity down to single particles,^11^ but require cryogenic conditions and complicated electronics. Magnetic force microscopy (MFM) is comparatively simple and can measure the magnetic properties of single particles, yet it is limited by drawbacks such as topographic cross talk or magnetic distortion by the scanning probe’s stray field. The recently developed single-particle PT MCD^12^ provides high spatial resolution with a simple table-top optical setup and has already shown great promise in studying the magneto-optical properties of single nanoparticles down to a size of 20 nm.

## Basic Principle of Single-Particle PT CD Microscopy

PT CD microscopy is based on the principle of photothermal (PT) microscopy.^13^ A modulated heating laser illuminates a nanoparticle that absorbs the light and then nonradiatively releases heat into the surrounding medium. The temperature profile surrounding the nanoparticle (under steady-state illumination, a *1/r* profile, where *r* is the distance from the center of the particle) creates a similar *1/r* refractive index change due to the thermo-refractive properties of the medium.^14^ The refractive index profile acts as a lens (a divergent lens called the thermal lens). A second beam is used to probe the thermal lens. The probe beam is scattered by the nanoparticle and by the thermal lens, and the scattered beam interferes with the probe beam or its reflection, acting as a reference. Modulation of the heating laser power creates a modulation of the thermal lens and thereby of the interference signal at the modulation frequency of the heating laser. This weak signal is then detected sensitively using a lock-in amplifier. The lock-in signal is the photothermal signal, which is linearly proportional to the absorption cross section, heating and probe laser powers, and to the thermo-refractive coefficient of the medium as indicated by the following equation:
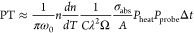
where PT is the photothermal signal, and *σ*_abs_ is the absorption cross section of the particle. *n*,  and *C* are the refractive index, thermo-refractive coefficient, and heat capacity of the surrounding medium, respectively. *P*_heat_ and *A* are the power and cross sections of the heating beam, respectively. *P*_probe_ and ω_0_ are the laser power and beam radius of the probe beam, respectively. Ω is the modulation frequency, and Δ*t* is the integration time.

**Figure 1:**
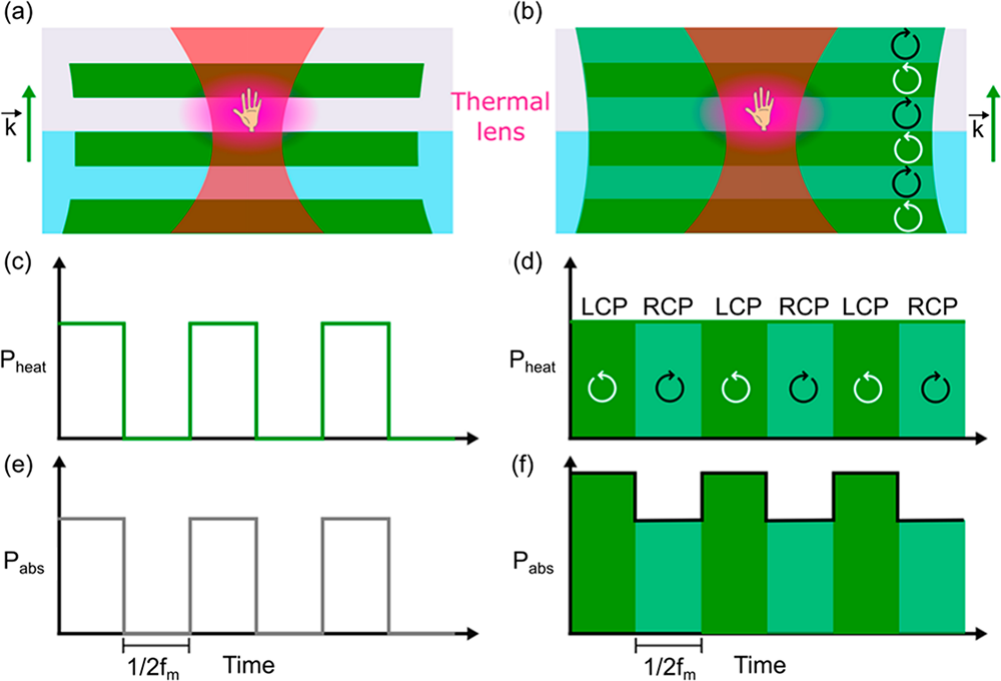
Schematic representation of photothermal (PT, left) and photothermal circular dichroism (PT CD, right) concepts. (a,b) The heating beam (green) illuminates a chiral nanoobject (represented as a hand) with a low NA, creating a wide-field illumination. The heat released from the nanoobject creates a thermal lens (purple), which is probed with a probe beam (red) focused on the sample with a high-NA objective. For photothermal detection (left), the heating beam is modulated in intensity whereas in photothermal circular dichroism (right), the heating beam is modulated between left- and right-circularly polarized light. The light propagation direction is denoted by the k-vector. (c, d) In the case of the standard photothermal mode, the heating laser power is modulated with a modulation frequency *f*_m_, while in the photothermal circular dichroism mode, the polarization is modulated at *f*_m_, while the heating laser power remains constant. Therefore, in the standard photothermal mode, the modulation of the absorbed power (e) is due to the modulation of the heating intensity, whereas, in the photothermal circular dichroism mode, the modulation of the absorbed power is due to the chiral nature of the particle. Depending on the handedness of the particle, the heating beam is absorbed by the particle differently for left- and right-circularly polarized light. The figure is taken from ref (4) with permission. Copyright 2019 American Chemical Society.

Instead of the intensity modulation of standard photothermal microscopy, in PT CD microscopy, the heating laser is modulated between left- and right-circular polarization, as schematically shown in Figure 1. Similar to the above equation, the PT CD signal can be written as follows:
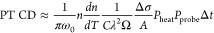
where Δσ is the differential absorption of left- and right-circularly polarized light. Most single-particle CD microscopy based on extinction or scattering uses a highly focused illumination to obtain a high spatial resolution; however, at a high numerical-aperture (NA) illumination, the polarization at the focus is prone to artifacts caused by even tiny misalignments.^15^ For example, any small mechanical drift in the focus can create a large polarization change that one could wrongly assign to a CD signal. To maintain polarization at the illumination focus (i.e., to reduce the sensitivity to misalignment in the focus), PT CD microscopy uses low-NA illumination of the heating beam. Importantly, to maintain a high spatial resolution in PT CD microscopy, the probe beam is strongly focused on the sample. In contrast to extinction- or scattering-based single-particle CD microscopy, PT CD microscopy requires careful polarization control of only one beam, the excitation beam, but it does not need to be concerned with the polarization of the detected probe light (polarization imperfections of the unpolarized probe light have no influence on the CD measurement).

Most chiral plasmonic nanoparticles have strong linear dichroism (LD) in addition to their CD. LD is usually orders of magnitude stronger than CD. Due to weak linear birefringence of polarizing optical elements (even nonpolarizing elements such as mirrors can distort polarization) in the excitation path, any imperfections in polarization can induce LD signals, which can be misinterpreted as CD. Therefore, LD needs to be carefully eliminated in CD detection. In addition, polarization modulators, such as electro-optic modulators (EOM) can create artifacts through residual intensity modulation between left- and right-circularly polarized light at the polarization modulation frequency.^16^ The residual intensity modulation can be misinterpreted as CD. To avoid the above-mentioned artifacts, PT CD microscopy uses dual polarization modulation (e. g., with an EOM and a photoelastic modulator, PEM operated at a different frequency). This approach eliminates any artifact produced at the individual modulators’ modulation frequencies. The PT CD signal can be detected at the sum frequency of the two modulators.^9^ The sum frequency was chosen instead of the difference frequency to reduce the contribution of the *1*/*f* noise. Using an EOM has the advantage that any polarization artifact can be balanced by slight offsets of the bias voltage in combination with optimization of the retardance of a quarter-wave plate. The technical details about the dual modulation PT CD setup can be found in ref (9).

## Basic Principle of PT MCD Microscopy

The main magneto-optical property exploited in PT MCD microscopy is the polar magneto-optical Kerr effect.^17^ When a magnetic nanoparticle is excited with light, light absorption due to spin–orbit coupling depends on the orientation of the magnetic moment with respect to the light polarization. The magnetic nanoparticle absorbs left- and right-circularly polarized light differently depending on the orientation of the magnetic moment with respect to the light propagation direction.^18^ The differential absorption of left- and right-circularly polarized light of a magnetic material in the presence of an external magnetic field or due to the intrinsic magnetic moment (i.e., for ferro- or ferrimagnetic material) is called magnetic CD (MCD).^19^ For a (super)paramagnetic object whose magnetization flips with the applied magnetic field, the MCD signal also flips with the field, thus providing a convenient signature of an MCD signal against static chiral signals due, for example, to the chiral shape of the object.

**Figure 2:**
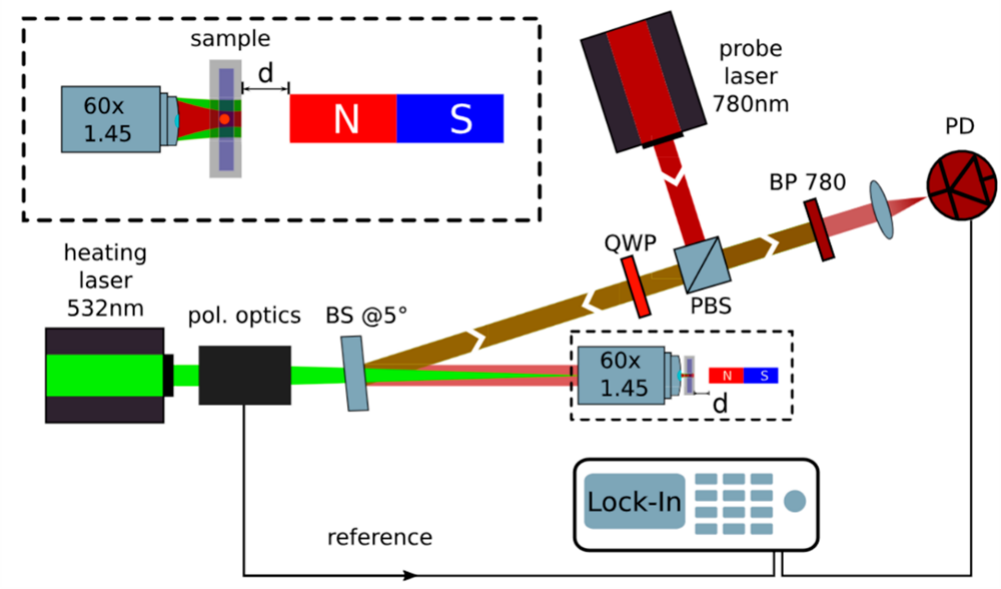
Schematic of the photothermal magnetic circular dichroism microscope setup. A 532 nm heating laser beam is passed through a combination of polarization optics (electro-optic modulator (EOM), photoelastic modulator (PEM) and a quarter-wave plate (QWP)) to modulate the heating laser between left- and right-circular polarization at ∼100 kHz. A 780 nm probe laser is passed through the combination of a polarizing beam splitter (PBS) and a quarter-wave plate (QWP) to make the probe beam circularly polarized. Both beams are combined at the 50/50 beam splitter (BS) at an angle of about 5°. The heating beam is focused at the back-focal plane of an oil-immersion objective (NA = 1.45) to make a Koehler illumination, whereas the collimated probe beam is tightly focused by the objective at the sample. The probe light is detected in the backward direction by using a photodiode after filtering the heating beam by using a band-pass filter (BP 780). The photothermal signal is measured using a lock-in amplifier. To apply the magnetic field, a permanent magnet is positioned parallel to the beam propagation direction, and to vary the field, the magnet is moved with respect to the sample. The inset shows an enlarged view of the magnet’s position relative to the sample. To invert the field direction, the magnet’s orientation is flipped. The figure is taken from ref (12) with permission. Copyright 2022 American Chemical Society.

As PT CD microscopy can measure the differential absorption of left- and right-circularly polarized light, incorporating an external magnetic field in the PT CD setup enables measurements of the magnetic circular dichroism signal. To apply an external magnetic field in PT MCD, we used a permanent neodymium magnet, but an electromagnet would also be suitable. One needs to check carefully that the polarization properties of optical components, particularly of the microscope objective, are not influenced by the applied magnetic field. To vary the magnetic field, the distance of the permanent magnet from the sample is varied, and to reverse the magnetic field, the magnet orientation is flipped. The PT MCD setup is the same as the PT CD setup with the addition of the magnetic part. A schematic representation of the PT MCD setup is shown in Figure 2.

## Applications of Single-Particle PT CD Microscopy

### Nanofabricated Plasmonic Chiral Nanostructures

**Figure 3:**
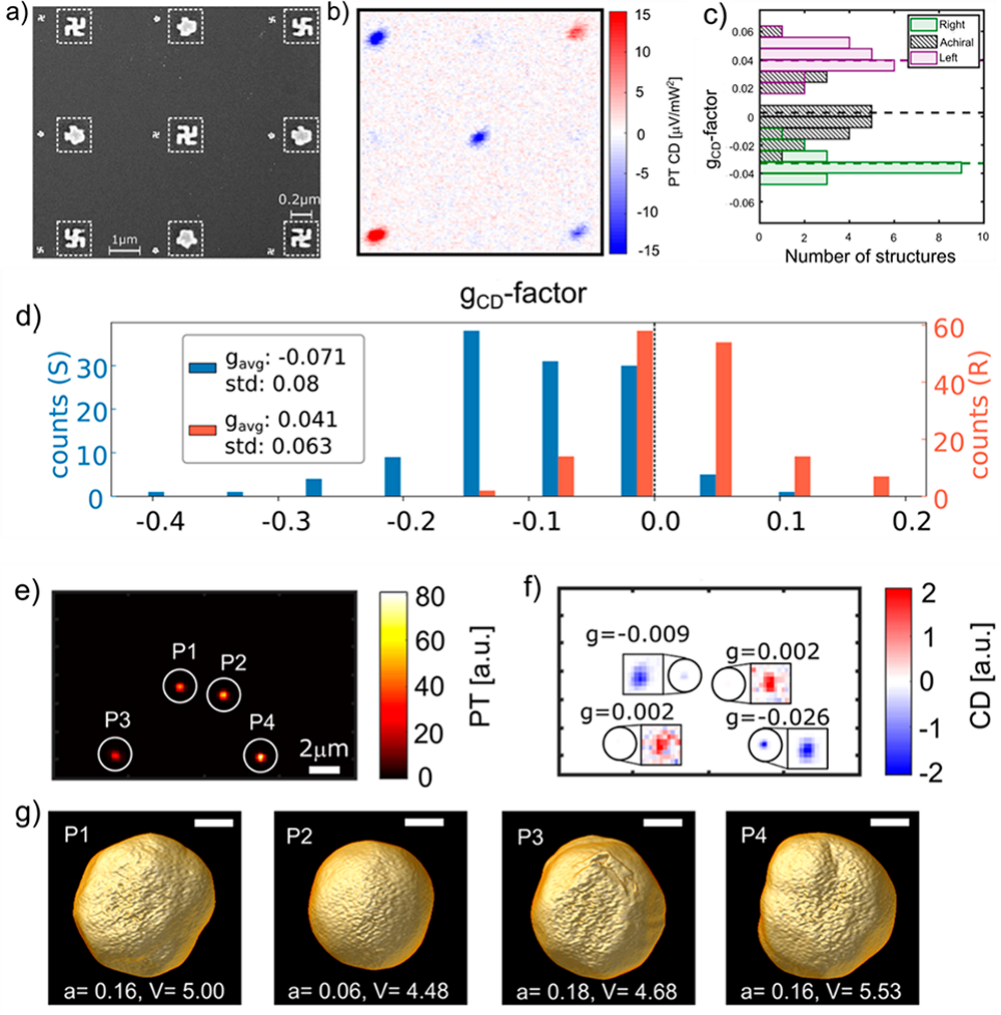
(a) Scanning electron microscopy (SEM) images of an array of left- and right-handed gammadion structures and achiral structures. (b) Corresponding photothermal circular dichroism (PT CD) images. PT CD images of single chiral nanostructures show good contrast and sign inversion with reversal of their handedness, while achiral structures show near-zero signal. (c) Histograms of g_CD_-factors of several chiral and achiral nanostructures showing the heterogeneity of single particles; however, the average g_CD_-factor is consistent with the ensemble value for each handedness. The panels (a–c) are taken from ref (4) with permission. Copyright 2019 American Chemical Society. (d) histograms of g_CD_-factors of single chiral nanorods from two types of enantiomers (R and S). The broad distribution indicates the heterogeneous chiral properties of single chiral nanorods, to the extent that several individuals show an opposite chiro-optical response from the bulk’s one. The figure is adapted from ref (24) with permission. Copyright 2022 American Chemical Society. (e) Photothermal (PT) and (f) circular dichroism (CD) images of four seemingly achiral gold nanoparticles. In (f), each image spot is adjusted to show better contrasts as shown in the inset. g_CD_-factors are also mentioned in the inset. (g) three-dimensional electron tomography images of the same four particles shown in (e) and (f). Asphericity (unitless) and volume (in nm^3^) are given in the inset. The scale bar is 50 nm. The figure is adapted from ref (24) with permission. Copyright 2022 American Chemical Society. Note that the SEM image in (a) may be viewed as potentially offensive. However, the gammadion motif is used as a standard in chirality studies.^2^

Several research groups recently focused on the design and nanofabrication of chiral plasmonic nanostructures using top-down lithography.^20^ These nanostructures usually show strong chiral signals, for example, gammadion structures (with C4 symmetry).^21^ Single-particle extinction- or scattering-based methods have shown imaging of CD signals of single gammadion structures.^2^ As discussed above, PT CD microscopy enables measuring direct differential absorption of left- and right-circularly polarized light by a chiral nanoobject. Spaeth et al.^4^ demonstrated PT CD imaging of single gammadion nanostructures. They nanofabricated an array consisting of left-handed, right-handed gammadions, and achiral structures (Figure 3a). Figure 3b shows a PT CD image of such an array, which demonstrates the signature of CD imaging, i.e., the flip of CD sign with reversal of the handedness of the chiral structures while achiral structures show near-zero signal. As imperfections in nanofabrication cause heterogeneity on individual nanostructures, the PT CD signals of a number of particles showed a broad distribution of g_CD_-factors (Figure 3c). The g_CD_-factor is the CD signal normalized by the total absorption (i.e., the photothermal signal as shown in Figure 3b) and defined as follows:
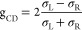
where *σ*_L_ and *σ*_R_ are the absorption cross sections of left- and right-circularly polarized light, respectively, by a chiral object. We use the g_CD_-factor term instead of the g-factor to avoid any confusion with the standard notation in atomic physics for the Landé factor. The g_CD_-factors for individual gammadion structures were about a few 10^–2^. The detection sensitivity reached up to 4 × 10^–3^ with an integration time of 30 ms, which was more than an order of magnitude improvement compared to previous demonstrations of a single-particle CD based on extinction or scattering. Later studies^9^ using dual polarization modulation showed that the detection sensitivity of PT CD could reach a few 10^–4^.

### Chemically Synthesized Chiral Gold Nanorods

In contrast to nanofabrication, chemical synthesis can mass-produce chiral nanostructures.^22^ Several research groups have tried to synthesize chiral structures with a high g_CD_-factor. Very recently, Lee et al. reported plasmon-driven synthesis of chiral nanoparticles via chirality transfer from circularly polarized light, without using any chiral molecules.^23^ Most studies focused on characterizing those nanoparticles at the ensemble level but were unable to explore their heterogeneity at the single-particle level. In addition, ensemble CD spectroscopy cannot directly correlate the morphological features of single particles to their bulk optical properties. Spaeth et al.^24^ reported single-particle PT CD imaging of chemically synthesized chiral gold nanorods. These nanorods had chiral wrinkles on the surface which were considered as the main reason for their chiroptical properties.^25^ Ensemble CD spectroscopy showed the sign inversion of two opposite enantiomers of these chiral nanorods. Figure 3d shows the distributions of single-particle PT CD signals of those two enantiomers. The mean values of the PT CD signal for both R and S enantiomers match the ensemble measurements. In addition, single-particle PT CD measurements showed that chiral nanorods were heterogeneous at the single-particle level; the inhomogeneous distribution exceeded the average value so that a significant number of nanorods showed opposite signs compared to their ensemble CD. A correlative study of PT CD imaging and three-dimensional electron tomography of single chiral nanorods attempted to find a correlation between morphological features and chiroptical properties.^24^ It was found that the features of chiral wrinkles might be related to the CD signal of single chiral nanorods; however, some particles showed a strong CD signal even without any obvious structural chiral features. The local chiral features might contribute strongly to the chiroptical nature of those particles. Further investigation is needed to understand the correlation between the structural features of those chiral nanostructures and the CD signal.

### Seemingly Achiral Gold Nanoparticles

Gold nanoparticles are usually considered achiral, because they are presumed to have a spherical shape. However, chemically synthesized nanoparticles are, in general, heterogeneous. Structural heterogeneity, such as shape, surface features, and crystal defects can induce chiroptical signal in a single nanoparticle. Spaeth et al.^24^ performed PT CD imaging of single chemically synthesized gold nanoparticles, which showed heterogeneous CD signals with positive and negative signs, as shown in Figure 3f. The mean value of the distribution was found to be near-zero, indicating the achiral nature of those particles at the ensemble level; however, at the single-particle level, some nanoparticles showed a very strong CD signal, with g_CD_ factors of more than 10^–2^. To understand the relation between a particle’s structural features and its chiroptical properties, these authors performed a correlative study of PT CD spectroscopy and 3D electron tomography of single gold nanoparticles (Figure 3e–g). It is evident that if the particle has a near-spherical shape, that particle would show a near-zero CD signal. However, if the particle has a nonspherical shape, it is difficult to predict its chiroptical response from its geometry. Boundary element analysis of those nanoparticles also predicted similar behavior. Future studies are needed to understand the origin of the chiral signal of these single metal nanoparticles.

## Applications of Single-Particle PT MCD Microscopy

### Superparamagnetic Nanoparticles

Magnetic nanomaterials have shown promising applications in data storage, fast computing, and spintronics. While technology requires smaller and smaller nanoparticles, the magnetic properties of smaller nanoparticles are affected by the so-called superparamagnetic limit. When the magnetic anisotropy energy barrier of a nanoparticle is comparable to the thermal energy, the magnetic spin can spontaneously switch between two opposite spin states over the experimental time scale so that the particle shows no net magnetic moment on average. Just like paramagnetic particles, superparamagnetic particles can be magnetized by applying an external magnetic field; however, their magnetic susceptibility is much higher than that of paramagnetic particles. PT MCD imaging^12^ of clusters of superparamagnetic magnetite nanoparticles (called nanoparticulate clusters) containing several thousands of 8 to 13 nm magnetite nanoparticles demonstrated their superparamagnetic behavior at the level of single nanoparticulate clusters. The high contrast in the PT MCD signal enabled measurements of a full magnetization curve. The g_CD_-factor of MCD is related to the magnetic susceptibility of the nanoparticle, i.e., the magnetic moment normalized by the particle’s volume and the applied field. The magnetic moment of a superparamagnetic particle can be obtained by fitting the magnetization curve with the following Langevin equation corresponding to a continuous distribution of magnetic moment orientations:
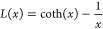
where , μ is the magnetic moment, *B* the applied magnetic field, *k*_B_ the Boltzmann constant and *T* the absolute temperature. The magnetic moment of a single magnetite nanocluster obtained from the PT MCD imaging matched the value obtained from the ensemble experiment, i.e., about 10^4^ Bohr magnetons.

### Synthetic Antiferromagnetic Nanoplatelets with Perpendicular Magnetic Anisotropy

Synthetic antiferromagnetic (SAF) nanoplatelets with perpendicular magnetic anisotropy (PMA) have great potential for nanoscale mechanical torque transfer in biomedical applications.^26^ These nanostructures have two ferromagnetic layers which are antiferromagnetically coupled, and thus, in the absence of an external magnetic field, their net magnetization is zero. However, upon application of a high enough magnetic field, the two layers couple ferromagnetically, thereby creating a large magnetic moment, which can be driven under modulation of the applied magnetic field direction. The dissipated heat can be used for cancer treatment in so-called magnetothermal therapy.^26^ The switching fields at which the nanoplatelets become ferromagnetic are important parameters in controlling their magnetization reversal. Ensemble studies have found that these switching fields are broadly distributed, which has been explained by a stochastic thermally activated process of small domain nucleation formation and subsequent domain-wall propagation.^27^ However, there were no direct studies of single nanoparticles. PT MCD imaging revealed the magnetic behavior of these nanoplatelets at the single-particle level.

**Figure 4:**
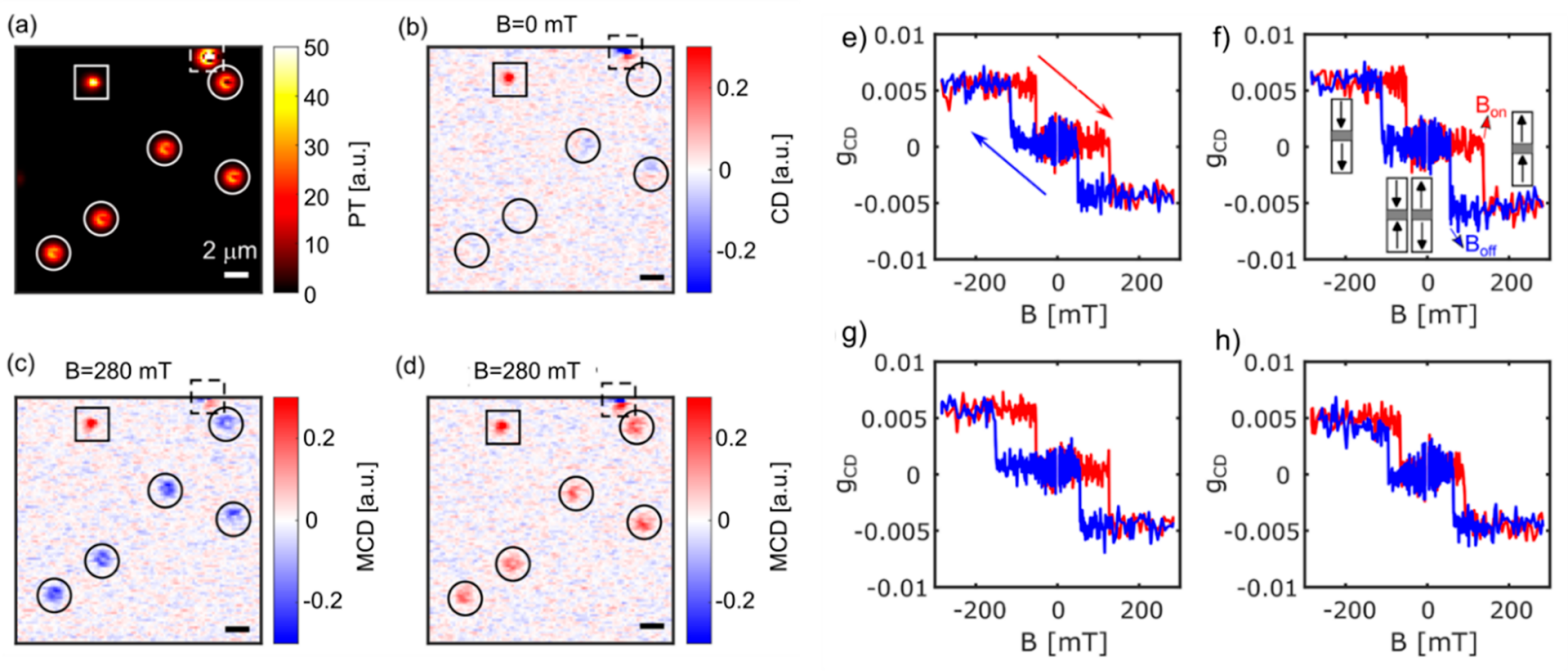
Single magnetic nanoplatelets: (a) photothermal (PT) image, (b) circular dichroism (CD) image without any applied external magnetic field, (c) magnetic circular dichroism (MCD) image with an applied magnetic field of 280 mT, and (d) MCD with an applied magnetic field of −280 mT. The magnetic nanoplatelets (solid circles) are 120 nm in diameter and 15.2 nm in thickness. The MCD signal flips its sign with an inversion of magnetic field direction, providing a convenient signature of MCD signals. Aggregates and nonmagnetic particles are shown with dashed and solid squares, respectively. (e–h) magnetization curves of four single magnetic nanoplatelets showing heterogeneity in their magnetization switching fields. The figure is adapted from the ref (28) with permission. Copyright 2023 American Chemical Society.

Adhikari et al.^28^ reported single-particle PT MCD measurements of single 120 nm SAF-PMA nanoplatelets, as shown in Figure 4. PT MCD allowed these authors to measure the magnetization curves of single nanoplatelets. In the absence of an external magnetic field, the PT MCD signal of those nanoplatelets showed an antiferromagnetic behavior, whereas in the presence of a magnetic field, the PT MCD signal showed the characteristic magnetization switching of the nanoplatelets. In contrast to ensemble studies of these particles, single-particle hysteresis loops showed sharp magnetization switching, in agreement with the domain-wall propagation mechanism. This was a first-time demonstration of such magnetization switching at the single-particle level. Single-particle PT MCD showed heterogeneity in magnetization switching fields from particle to particle, which was predicted by the slant of the magnetization curve reported in the previous ensemble experiments. Single-particle PT MCD imaging showed direct evidence of a large switching field distribution (SFD). In addition to the spatial heterogeneity among different nanoplatelets, PT MCD made it possible to measure a single nanoplatelet over several repeated hysteresis cycles. The switching field was reported to vary over time due to a stochastic thermally activated nucleation process. Such temporally heterogeneous magnetization switching was reported for the first time for single magnetic nanoparticles. Single-particle PT MCD microscopy has great potential for future studies of these SAF-PMA nanoplatelets for magnetothermal therapy applications in biological systems.

### 20 nm Magnetite Nanoparticles

**Figure 5:**
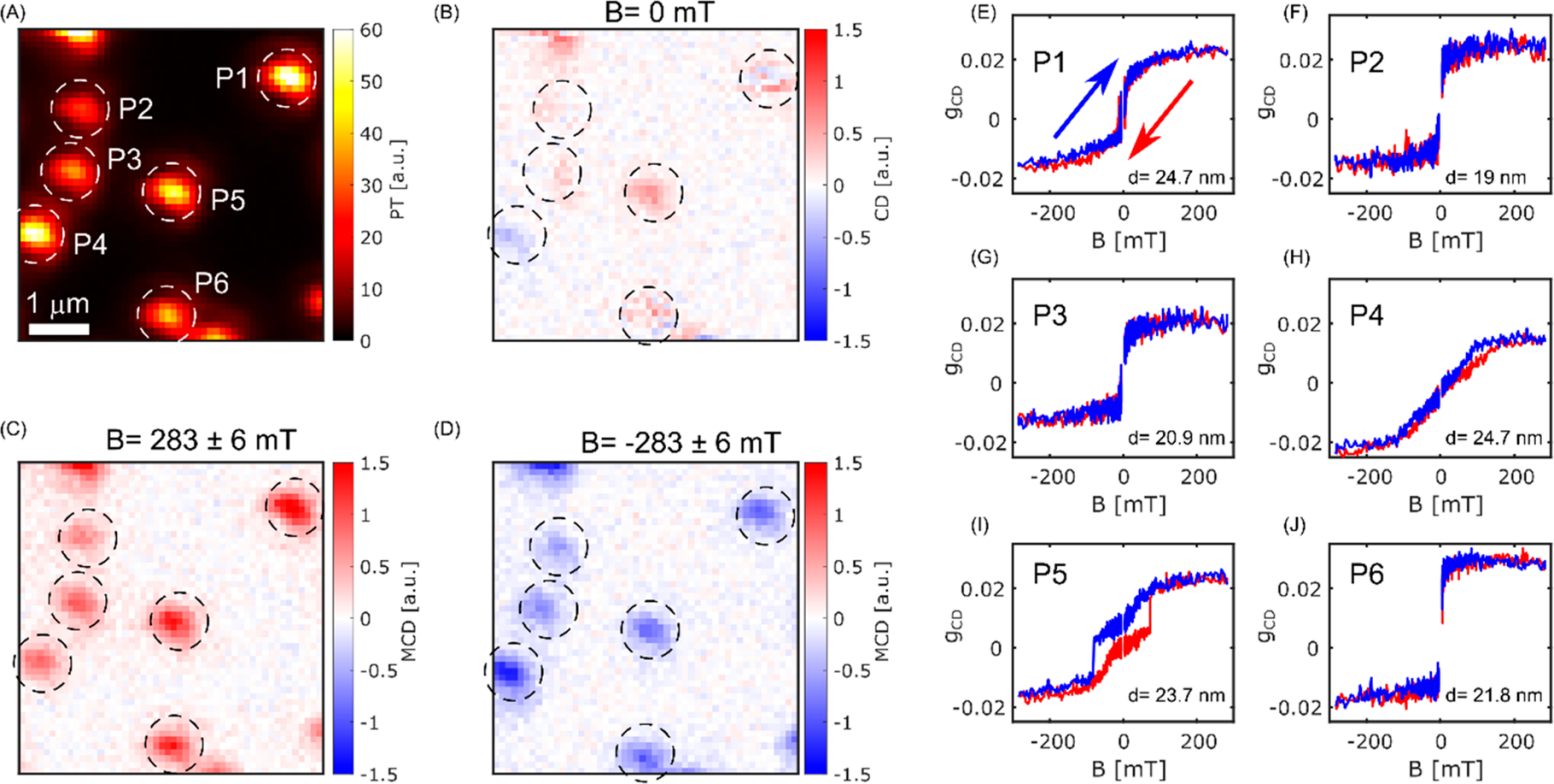
Single 20 nm magnetic nanoparticles: (A) photothermal (PT), (B) circular dichroism (CD), (C) MCD images at 283 mT and (D) MCD images at −283 mT. Their corresponding magnetization curves are shown in (E–J). Particle P1 shows spontaneous switching at zero field, and particle P5 shows ferrimagnetic properties. Particles P2, P3, P4 and P6 show superparamagnetic properties while their saturation behavior and magnetization curve vary from particle to particle. The figure is taken from the ref (29).

Single-particle PT MCD demonstrated its high detection sensitivity by imaging the magnetization of single 20 nm magnetite nanoparticles, with a detection sensitivity of a few 10^4^ Bohr magnetons.^29^ These single-domain nanoparticles were found to be highly heterogeneous in their magnetic properties, depending on their size, shape, and orientation with respect to the applied magnetic field. Single-particle PT MCD magnetization curves, as shown in Figure 5 displayed superparamagnetic to ferrimagnetic behavior, thereby providing information about their shape anisotropy and orientation according to the Stoner–Wohlfarth model.^30^ Adhikari et al.^29^ reported that some of these nanoparticles showed spontaneous magnetization switching in the absence of an external magnetic field. This was the first demonstration that one could track spontaneous magnetization switching optically. The thermally induced switching times varied from milliseconds to minutes. Such a temporal heterogeneous behavior, the so-called dynamical heterogeneity, is a well-known phenomenon in glass-forming liquids^31^ and in protein dynamics.^32^ Magnetic-field-dependent single-particle magnetization switching rates enabled the determination of the magnetic moment of single magnetite nanoparticles, and the temperature dependence gave access to the magnetic anisotropy energy barrier of single magnetite nanoparticles. These 20 nm magnetite nanoparticles were found to have a magnetic moment of about a few 10^5^ Bohr magnetons and an anisotropy energy barrier of about 0.8 eV. The temporal change of magnetization switching rates is not yet well understood and remains an open question for future studies.

## Perspectives

### Photothermal Circular Dichroism Spectroscopy

PT CD studies of single (plasmonic) nanoparticles have shown that ensemble CD properties can strongly differ from single-particle CD properties. Particles that were assumed to be achiral showed weak chirality, and tailored chiral nanoparticles showed a broad range of circular dichroism strength, including an opposite sign from the ensemble. Additionally, to study more complex particles such as the ones reported in ref (25), spectroscopic information is imperative. CD signals of plasmonic nanoparticles often change sign depending on the measured wavelength. Such changes in CD sometimes can be quite sharp. Therefore, a particle can show weak to no CD signal at one wavelength, whereas it can show strong CD signals at slightly shifted (red or blue) wavelength. PT CD has so far been demonstrated only for single wavelengths,^9^ but in principle, with tunable lasers or with strong enough white-light sources (∼several mW per nm), PT CD can be turned into a spectroscopic technique. Although PEM’s are typically used in polarimeters due to their longer lifetime and ease of use, EOM’s have the advantage that the chromatic retardance of the necessary quarter-wave plate can be easily compensated by applying a wavelength-dependent bias electric field. PT CD spectra can help understand the exact relationship between shape and circular dichroism, for example, by combining CD spectroscopy with electron tomography and therefore help improve the design of novel chiral materials. PT CD spectroscopy can also be extended to time-resolved studies of chiral nanostructures.^33^

### Sensing Molecular Chirality Using PT CD

Biomolecules exhibit electronic CD in the UV region (200–300 nm), typically in the range of millidegrees and vibrational CD in the (mid) IR regime is about 2 orders of magnitude smaller.^34^ Therefore, both CD signals are much weaker than the CD of chiral plasmonic nanoparticles, which can exhibit CD as high as hundreds of millidegrees. In order to improve sensitivity in the detection of molecular chirality, several groups use approaches wherein chiral or achiral plasmonic nanoparticles boost the CD signal of chiral molecules both in the visible and mid-IR regime, by means of near-field enhancement or superchiral fields.^1^ However, such approaches usually make use of a large number of plasmonic structures to compensate for the contribution of the plasmonic particles themselves to the overall CD signal. Although some of these ensemble methods achieve great sensitivity, they inevitably suffer from poor spatial resolution. A recent work attempted to detect an enhanced CD signal using PT CD microscopy of a single plasmonic nanoparticle, but showed no clear enhancement of the CD signal.^9^ There are several reasons for this: first, the chosen chiral molecules (carvone) had lower CB effect at the measured wavelength, whereas chiral molecules such as binaphthyl would present stronger CB effects; second, as plasmonic nanoparticles are very strong absorbers, the use of higher laser intensities to enhance the sensitivity would result in severe heating effects and photochemistry close to the nanoparticles; third, even supposedly achiral plasmonic particles can exhibit intrinsic CD due to shape irregularities. A more promising approach to using PT CD in sensing molecular chirality would be to use nonabsorbing dielectric nanoparticles as a means to strongly enhance the field and to limit the excitation of chiral molecules to a small volume. Chiral molecules in the hot spot of such structures would undergo enhanced absorption, whereas the intrinsic absorption of the dielectric nanoparticles could remain low, in comparison to plasmonic ones. Both the damage due to heating and the intrinsic CD signal contribution of the dielectric particles would be lowered.

### Photothermal Vibrational Circular Dichroism (PT VCD) Spectroscopy

Another approach to sensing molecular chirality via PT CD would be to use vibrational absorption. In vibrational absorption spectroscopy, molecular bonds are excited directly. As stated above, the effect of VCD of molecules is typically much weaker than CD in the UV regime,^34^ but the benefit is that vibrational excitation does not lead to photodamage of the molecules. Photothermal VCD can also be combined with plasmonic or dielectric nanoparticles, where the plasmonic or dielectric particles themselves (no longer the thermal lens) might act as thermal transducer. When probing the nanoparticle on the wing of the plasmon (typically in the visible) while heating the molecules via VCD, signals could be much stronger than those in PT CD based on a thermal lens. The LSPR (localized surface plasmon resonance) of plasmonic nanorods or the Mie modes of dielectric particles are very sensitive to the refractive index in the near field;^35^ therefore changes in this refractive index due to differential absorption of molecules will shift the sharp resonances and can be exploited to enhance the signal. Larger dielectric nanoparticles or carefully designed nanoparticle assemblies/clusters with multiple resonances could also be used in a 2-fold manner: at one wavelength, they could provide a near-field for enhanced absorption of chiral molecules, and at a second wavelength, they could be used to sensitively probe the refractive index change induced by molecular differential absorption. Recent work using supercritical CO_2_ for photothermal detection^36^ has shown great improvement in sensitivity for PT imaging and could be combined with the aforementioned approaches, but the use of CO_2_ would limit biochemical applications.

### Application of PT MCD in Magnetic Hyperthermia

The heating of local media surrounding an embedded magnetic nanoparticle (MNP) via an applied alternating magnetic field in magnetic hyperthermia, similar to inductive heating, holds great promise for efficient in situ heating methods for industrial and biomedical applications.^37^ One of the main limitations of magnetic hyperthermia is the quantification of heating power expressed in the specific absorption rate (SAR) or the specific loss power (SLP), i.e., the normalized (by mass) rate of energy dissipation caused by an alternating magnetic field. Despite many standardization efforts, the reported SAR values vary widely among laboratories for nominally identical MNPs. It is conjectured that this discrepancy is due to the substantial heterogeneity of the used MNPs, both in size/shape and magnetic properties, which determines the fundamental SAR.^38^ PT MCD can be used to study the SAR at the single-particle level. A better control of magnetic hyperthermia of ensembles would thus benefit from the development of measurement methods for single MNP’s. Despite considerable progress in the simulation of individual particle heating, the corresponding experimental observations are still missing.

The potential of single-particle hyperthermia is illustrated by several examples. For instance, it is generally considered that homogeneous assemblies of MNPs with narrow size distribution can be heated more efficiently in an external magnetic field compared to heterogeneous ensembles. Nevertheless, other reports^39^ claim that combining different MNPs (different materials and core–shell combination and size) can lead to the synergetic increase of the total SAR value, which cannot be understood from the macroscopic observations. Another prominent example is unravelling the potential of the MNPs chains in magnetosomes of magnetotactic bacteria for magnetic hyperthermia *in vivo*,^40^ where the MNPs size and the distance between the MNPs in the chain significantly influences the effective SAR value. However, it was not possible to experimentally measure the heating of a single magnetosome, and most of the studies are performed either in ensembles or computationally.

### Investigation of Biogenic Magnetic Materials

PT MCD can be used to study biogenic magnetic materials such as magnetotactic bacteria, tissues of animals possessing magnetoreception, and human brain tissues. There, nondestructive magnetometry techniques combined with an imaging modality to characterize the (element-specific) response from very small (down to 1–10 nm) nanoparticle assemblies located in a predetermined part of the cell or tissue are in high demand. Currently, investigation of such samples often involve synchrotron radiation with X-ray Magnetic Circular Dichroism (XMCD) being the most common technique.^41^ In XMCD, one uses the differential absorption of left- and right-circularly polarized X-ray light in a magnetic field to examine magnetic materials. In this case, the “complementary” imaging technique is scanning transmission X-ray microscopy (STXM). Synchrotron radiation-based techniques offer a high spectral resolution and thus a high elemental specificity^41^ allowing to determine the oxidation state of magnetic ions. However, synchrotron-based methods have a substantial disadvantage, namely, the radiation sample damage due to the high energy of X-rays destroying the cells/tissues. The radiolysis of water, lipids and other components produces various radicals and ions that interact with magnetic materials and change their properties–in particular, the reduction of Fe(III) to Fe(II) as a result of radiation damage leading to the measurement artifacts is well documented in the literature.^42^

PT MCD spectroscopy, similar to the above-mentioned PT CD spectroscopy, could be an alternative to the XMCD/STXM for characterizing sensitive and “weakly” magnetic materials from biological samples. For example, one may study the properties of chains in magnetotactic bacteria given their potential for magnetic hyperthermia,^43^ inspect the biomineralization pathways of Fe in magnetotactic bacteria and chitons (monitoring the process of conversion of antiferromagnetic ferritin into ferrimagnetic magnetite) as well as the alteration of Fe metabolism in the human brain leading to various neurodegenerative disorders including Alzheimer’s disease. If the combination of PT MCD with the ferromagnetic resonance (FMR) modality is possible (as discussed below), monitoring magnetic resonance modes within chains of particles in magnetotactic bacteria for biologically encoded magnonic devices^44^ may be one of the appealing perspectives. Last but not least, a PT MCD setup can be assembled in a “standard” optical lab and does not require synchrotron radiation facilities.

### Photothermal Detection for Enhanced Resolution in Magnetic Property Characterization: Integrating Radio Frequency (RF) Antennas for Nanoparticle Analysis

Photothermal detection techniques have facilitated the advancement of magnetic property characterization by offering enhanced depth and lateral resolution capabilities. This is predominantly achieved through the localized heating and modulation of magnetic properties by using a focused laser beam in combination with conventional microwave methodologies for FMR detection. Such an approach has demonstrated the potential to provide spatial resolutions in the range of 10 to 100 nm.^45^ The extension of this approach to incorporate PT MCD holds promise for unprecedented insights into the magnetic properties of nanoparticles by leveraging the information derived from FMR analysis.

To further advance this methodology, a pivotal development might be found in the integration of strip line RF antennas into the sample holders used for PT MCD. This innovation allows for the application of variable RF fields within the frequency band pertinent to the FMR of the nanoparticles. Implementation of this concept can be realized through flip-chip-like techniques, as exemplified in previous works (see, for instance, ref (46)). At resonance conditions, the power absorbed by the magnetic nanoparticles can be sensitively detected through the photothermal signal, while the magnetic circular dichroism signal can provide additional insights into the FMR mode. This combined approach holds particular promise for accessing FMR-assisted thermal magnetization switching at the nanoscale, as demonstrated in prior research (see, for example, ref (29)).

### Spintronic Applications and Time-Resolved Magnetization Measurements

Research on ultrafast and highly energy-efficient switching of nanoscale magnetic structures has recently received growing attention. Aiming for Information and Communication Technology (ICT) with drastically reduced energy consumption, enhanced data rates, and a smaller footprint, novel ways to manipulate magnetic ordering and switch nanoscale bits are being searched for. In this endeavor, revolutionary schemes for magnetization switching by driving electric currents, but also just by sending single pulses of light, have been demonstrated. These developments heavily rely on local probes of small magnetic elements and their dynamics to investigate the switching current density or threshold laser energy, respectively. PT MCD could provide a quasi-static experimental tool in this domain, although it would require new developments to implement this technique in a condensed matter environment rather than the nanoparticles embedded in thermo-refractive liquids, as discussed before in this paper. An ultimate goal would be not only to measure the switched magnetic state in a quasi-static experiment but also to monitor the magnetization dynamics in a time-resolved fashion at a picosecond and shorter time resolution.

In order to address the ultimate limits of magnetization dynamics, pulsed-laser-induced excitation schemes have provided a wealth of fundamental insight over the past two to three decades, a field that is nowadays dubbed femtomagnetism. The most basic phenomenon is the ultrafast quenching of magnetic order in a ferromagnetic thin film within approximately 100 fs by heating it with a fs laser pulse.^47^ Also, it was discovered that GHz—and even THz—magnetization precession can be excited by these fs laser pulses and optically probed in the time domain.^48^ Finally, it was demonstrated that single fs laser pulses can entirely reverse the magnetization in ferrimagnetic alloys and synthetic ferrimagnetic multilayers systems, a phenomenon dubbed all-optical switching of magnetization (AOS).^49^ While this discovery triggered great interest because of its potential application in ICT, the underlying dynamics is more generic and could also be used for other systems and applications, including magnetic nanoparticles used for bioapplications. As an example, extrapolating on the nanoplatelets discussed earlier in this review, one might envision engineered platelets that can optically be switched between the magnetic on and off states with a single flash of light.

Time-resolved investigation of ultrafast magnetization dynamics is typically performed in a pump–probe scheme in which a first pump laser pulse excites the magnetic system, triggering the magnetization dynamics, after which the magnetic state is probed by a second (probe) pulse arriving at a (variable) delay time. A common approach is using an optical pulse to probe the magnetic state via the magneto-optical Kerr effect (MOKE), referred to as time-resolved MOKE (TR-MOKE).^47^ Other alternatives use, e.g., X-rays^49^ or photoexcited electrons to probe. An example of using TR-MOKE for measuring the precessional magnetization dynamics in ensembles of (Co/core)-(Pt-shell) particles has been reported by Bigot et al.^50^ Performing ultrafast magnetization dynamics of single particles or small structures with a high spatial resolution, down to tens of nanometers, is challenging. These studies require large-scale facilities, such as synchrotrons or free-electron lasers, and/or an ultrahigh vacuum environment, such as when performing photoelectron emission microscopy (PEEM). In this respect, a time-resolved extension of PT MCD (TR-PT-MCD) might provide an attractive alternative, albeit it would need a carefully designed scheme using sequences of multiple pulses.

One could envision TR-PT-MCD in a stroboscopic three-pulse scenario, which needs careful consideration to account for the temporal evolution of the optical lens. A first (femtosecond) pump pulse is used to trigger the magnetization dynamics. Second, a circularly polarized heating pulse arrives after a variable delay time. Via the MCD effect, the magnetic state of the nano-object at this specific arrival time is encoded in the strength of the optical lens that starts to be formed at this moment. A third pulse, which we call the probe pulse, measures the scattering by the thermal lens, as in regular PT MCD. To optimize the signal strength, its arrival time should be well-tuned. A too early arrival would mean that heat diffusion from the absorbing particle to its surroundings and thermal expansion of the fluid have not yet proceeded enough to fully develop the thermal lens. Waiting too long will mean that the lens will start fading out by a further distribution of heat over too large a volume. By repeating this three-pulse sequence at a certain repetition (rep.) rate *F*_p_ and applying a polarization modulation of the heat pulses at a rep. rate *F*_M_ ≪ *F*_p_, a dichroic signal can be picked up by a lock-in amplifier. Alternatively, the handedness of the pump pulses could be modified at half the pump repetition rate, *F*_M_ = 1/2 *F*_p_, i.e., the polarization toggles between left- and right-circular polarization every next shot. Independent of the exact choice, scanning the delay time between the heat- and pump-pulses will lead to a time-resolved trace of the magnetization dynamics.

Finally, we will discuss some further considerations for this three-pulse scheme. (i) Although advantages exist for using three different colors, it may be more practical to use the same wavelength for pump and heat pulses, as in standard “degenerate” TR-MOKE measurements. Yet, the probe pulses should certainly have a distinct wavelength to distinguish them from the scattered pump and heat pulses unless fast time-gating is applied. (ii) The choice of the most appropriate rep. rate depends on factors such as the required laser power, the need of avoiding too much accumulative heat (which weakens temporal contrast), and also the temporal dynamics of the optical lens. (iii) As a possible alternative to using a pulsed laser as a probe, one could consider using continuous wave (CW) detection. Using such a CW probe would result in a time-averaged effect of the thermal lens, its amplitude yet being a measure of the magnetic state at the arrival time of the heating pulse. Although this would reduce the amplitude of the dichroic scattering signal, it could provide an attractive simplification of the experimental setup. To conclude, although ultrafast TR-PT-MCD has never been attempted yet, by having discussed its relevance as well as a concrete possible implementation, we hope to fuel further developments toward the realization of this appealing option.

Single-particle photothermal circular dichroism and photothermal magnetic circular dichroism microscopy have demonstrated great potential for applications in chiral plasmonics and the study of magnetic nanoparticles. Single-particle measurements enable the investigation of spatiotemporal heterogeneities at the nanoscale. This resulted in observations of the dynamical heterogeneity of a single antiferromagnetic nanoplatelet or of a single 20 nm magnetic nanoparticle. These promising techniques open the way to studies of many more plasmonic and magnetic nanomaterials and their applications in biology, from chiral molecule sensing to magnetic hyperthermia or to investigating biogenic magnetic materials. In material science and magnetism, ferromagnetic resonance studies and ultrafast time-resolved magnetization studies of a single magnetic nanoparticle would benefit many spintronics applications. We envision that this new field of optical probing of single magnetic particles will have a great impact on future research.
